# The Proosteogenic and Proangiogenic Effects of Small Extracellular Vesicles Derived from Bone Marrow Mesenchymal Stem Cells Are Attenuated in Steroid-Induced Osteonecrosis of the Femoral Head

**DOI:** 10.1155/2020/4176926

**Published:** 2020-05-06

**Authors:** Jia Li, Zhaogang Ge, Wenchen Ji, Na Yuan, Kunzheng Wang

**Affiliations:** ^1^Department of Orthopedics, The First Affiliated Hospital of Xi'an Jiaotong University, Xi'an, Shaanxi Province 710061, China; ^2^Department of Joint Surgery, Honghui Hospital of Xi'an Jiaotong University, Xi'an, Shaanxi Province 710054, China; ^3^Department of Ultrasonography, The First Affiliated Hospital of Xi'an Jiaotong University, Xi'an, Shaanxi Province 710061, China; ^4^The First Department of Orthopedics, The Second Affiliated Hospital of Xi'an Jiaotong University, Xi'an, Shaanxi 710004, China

## Abstract

Small extracellular vesicles (sEVs) derived from bone marrow mesenchymal stem cells (BMMSCs) from individuals with steroid-induced osteonecrosis of the femoral head (ONFH) have not been studied. The objective of the present study was to compare the proosteogenic and proangiogenic effects of sEVs derived from BMMSCs from rats with steroid-induced ONFH (oBMMSCs-sEVs) and sEVs derived from BMMSCs from normal rats (nBMMSCs-sEVs). BMMSCs were isolated from steroid-induced ONFH rats and healthy rats. sEVs were isolated and characterized by Western blotting analysis of exosomal surface biomarkers and by transmission electron microscopy. The impacts of nBMMSCs-sEVs and oBMMSCs-sEVs on the proliferation and osteogenic differentiation of BMMSCs were determined via cell proliferation assay, alizarin red staining, and alkaline phosphatase activity assay. Enzyme-linked immunosorbent assay and tube formation assay were conducted to investigate the effect of nBMMSCs-sEVs and oBMMSCs-sEVs on the angiogenic potential of human umbilical vein endothelial cells (HUVECs). The expression of relevant genes was detected by quantitative real-time polymerase chain reaction analysis, and the expression of *β*-catenin was detected by immunofluorescence. Both nBMMSCs-sEVs and oBMMSCs-sEVs promoted proliferation, osteogenic differentiation, and *β*-catenin expression of BMMSCs and enhanced angiogenesis of HUVECs. However, compared with nBMMSCs-sEVs, oBMMSCs-sEVs exhibited attenuated effects. Our findings indicated that the proosteogenic and proangiogenic effects of sEVs were partially attenuated in steroid-induced ONFH. Therefore, this study might offer guidance for the selection of source cells for sEV therapy in the future.

## 1. Introduction

Steroid-induced osteonecrosis of the femoral head (ONFH) is a refractory and progressive disease caused by glucocorticoid administration. Most of the affected patients are young people aged 30–50. If treatment is not properly administered in a timely manner, it will lead to articular cartilage collapse and secondary osteoarthritis, necessitating artificial joint replacement. These conditions cause a heavy burden on society and families. However, to date, the detailed pathological mechanism of steroid-induced ONFH is not clear.

Hypotheses about ONFH pathogenesis indicate that lipid metabolism disorders, hypercoagulable conditions, intraosseous hypertension, bone cell apoptosis, immune factors, and enhanced vasoconstriction are involved in the mechanisms of steroid-induced ONFH [1]. Moreover, dysfunction of the vascular endothelium and altered characteristics of mesenchymal stem cells have been suggested to contribute to the development of steroid-induced ONFH. Feng et al. have found that circulating endothelial progenitor cells (EPCs) from ONFH patients show decreased migratory abilities and attenuated in vitro angiogenic capacities compared with EPCs from healthy controls [2]. Previous studies and our research have found that bone marrow mesenchymal stem cells (BMMSCs) derived from osteonecrosis patients and animals exhibit attenuated survival and proliferation abilities and imbalanced osteogenic/adipogenic differentiation [3–6].

BMMSCs are multipotential cells that can promote tissue regeneration and repair. Recent studies have indicated that BMMSCs produce marked effects via the secretion of paracrine factors, including growth factors, cytokines, chemokines, and extracellular vesicles (EVs), into their surroundings [7]. EVs are lipid bilayered vesicles that are secreted from cells towards the extracellular space and play various roles in intercellular communication [8]. According to their size, origin, and isolation methods, EVs can be classified into three main groups: (i) exosomes: vesicles derived from the endosomal membrane with sizes of 30-120 nm in diameter; (ii) microvesicles: vesicles derived from the plasma membrane with sizes of 50-1000 nm in diameter; and (iii) apoptotic bodies: vesicles derived from fragments of dying cells with sizes of 800-5000 nm [9]. Among these paracrine factors, exosomes are attracting increasing attention given their important role in cell-cell communication. Evidence has demonstrated that exosomes can mostly recapitulate the regenerative activities of their parent cells [10]. Exosomes, secreted by most cell types, are cell-released membrane vesicles ranging in size from 40 to 100 nm. Exosomes are formed by the inward budding of the endosomal membrane, leading to the formation of intraluminal vesicles called multivesicular bodies (MVBs). MVBs can fuse with the plasma membrane to release exosomes to adjacent cells or into circulation [11]. Exosomes shuttle coding RNA, noncoding RNA, proteins, and other molecular constituents between cells to achieve cell-cell communication and exert biological regulatory impacts on recipient cells. Transplantation of exosomes from mesenchymal stem cells has demonstrated their potential for promoting tissue repair, including reducing myocardial infarction size [12], attenuating limb ischaemia [13], facilitating kidney injury repair [14], promoting neurological recovery [15], and enhancing bone regeneration [16, 17]. However, the different cell types, cellular environments, and pathological conditions of the parent cells affect the contents and functions of exosomes [18–20]. On the other hand, the compositions and biological activities of exosomes reflect the pathophysiological state of their parent cells. Qiao et al. found that the pathological condition of heart failure alters the miR cargos of cardiac-derived exosomes and impairs their regenerative activities [20]. Davidson et al. demonstrated that the cardioprotective effects of exosomes are impaired in both rats and humans with type 2 diabetes [21]. Azimi et al. indicated that the immunomodulatory function of exosomes derived from regulatory T cells is impaired in patients with relapsing-remitting multiple sclerosis [22].

According to the guidance of the International Society for Extracellular Vesicles (ISEV), the characterization of EVs includes the following: (i) demonstration of the presence of at least three positive protein markers of EVs (including at least one transmembrane protein and one cytosolic protein) and absence of at least one negative protein marker; (ii) single particle analysis by imaging techniques such as electron microscopy; (iii) single particle analysis by techniques (not electron microscopy-based) such as nanoparticle tracking analysis and flow cytometry; and (iv) supplementation by quantification of the total protein, lipids, or RNA [23]. Because of the difficulty in differentiating exosomes and microvesicles of the same size, in this study, vesicles smaller than 200 nm in diameter were defined as small EVs (sEVs) [24].

Considering that BMMSCs from animals and patients with steroid-induced ONFH show attenuated proliferation abilities and osteogenic potentials and that sEVs reflect the biological properties of their source cells, we hypothesized that sEVs derived from ONFH BMMSCs may have impaired biological activities. Hence, we investigated the in vitro proliferative, osteogenic, and angiogenic effects of sEVs derived from BMMSCs from rats with steroid-induced ONFH (oBMMSCs-sEVs) and sEVs derived from BMMSCs from normal rats (nBMMSCs-sEVs). The results may provide theoretical references for understanding the pathophysiology of ONFH and may offer guidance for the selection of source cells for sEV therapy in the future.

## 2. Materials and Methods

### 2.1. Establishment of the Animal Model of ONFH

Eighteen four-week-old male Sprague-Dawley rats from the Experimental Animal Center of Xi'an Jiaotong University (Shaanxi, China) were used. ONFH was established in nine rats according to a previously reported protocol [25]. Briefly, the rats were intravenously administered two injections of 4 mg/kg body weight lipopolysaccharide (LPS, Sigma, St. Louis, MO, USA) at a time interval of 24 h. Twenty-four hours later, the rats were given three injections of 60 mg/kg body weight methylprednisolone (Pfizer, New York, USA) at a time interval of 24 h. Four weeks later, the rats were sacrificed, and the ONFH BMMSCs were obtained for further study. The experimental protocols were approved by the Animal Ethical Committee of Xi'an Jiaotong University.

### 2.2. BMMSC Isolation and Culture

BMMSCs from healthy rats and ONFH rats were isolated according to a previously reported protocol [26]. Briefly, the femurs and tibias were obtained under sterile conditions after the rats were euthanized. The bone marrow was flushed out with 1 mL *α*-minimal essential medium (*α*-MEM, Gibco, CA, USA) and repeatedly washed. The obtained cells were seeded in 6-well dishes and cultured in *α*-MEM containing antibiotics (1% penicillin-streptomycin) and 10% fetal bovine serum (FBS, Gibco, CA, USA) in an incubator with 5% CO_2_ at 37°C. To remove the nonadherent cells, the medium was changed after 24 h. When the cells became subconfluent, the cells were passaged. Cells of passages 3-5 were used in subsequent experiments. The cell surface markers (including CD29, CD45, and CD90) were analyzed by flow cytometry (FACSCalibur; Becton Dickinson, NJ, USA).

### 2.3. Isolation and Characterization of nBMMSCs-sEVs and oBMMSCs-sEVs

After the BMMSCs reached 80-90% confluence, they were cultured in culture medium containing 10% exosome-free FBS for 48 h. Then, the culture medium was collected, and the BMMSC-derived sEVs were isolated by differential centrifugation. The conditioned medium was centrifuged at 300 × g for 10 min and 16500 × g for 20 minutes at 4°C. To further eliminate the cellular debris, the supernatant was filtered through a 0.22 *μ*m filter. The supernatants were then ultracentrifuged at 120000 × g for 70 min at 4°C to collect the sEVs. The sEVs in the pellet were resuspended in PBS and stored at -80°C. sEVs were collected from 5‐7 × 10^8^ BMMSCs (half were nBMMSCs and half were oBMMSCs, which were cultured in 80-100 T175 flasks in a total of approximately 2000 mL culture medium) and were used for the subsequent experiments. The sEVs collected from normal BMMSCs and ONFH BMMSCs were denoted as nBMMSCs-sEVs and oBMMSCs-sEVs, respectively. The protein content of the concentrated sEVs was measured using the BCA protein assay kit (Pierce, USA). Transmission electron microscopy (TEM) was performed to directly observe the size and morphology of the sEVs. Nanoparticle tracking analysis (NTA) was performed to analyze the absolute sEV size distribution by using NanoSight NS300 (Marvel, UK). In addition, the sEVs were identified by Western blotting with anti-CD9 (1 : 2000, rabbit monoclonal antibody, Abcam, USA), anti-CD63 (1 : 1000, mouse monoclonal antibody, Abcam, USA), anti-TSG101 (1 : 1000, mouse monoclonal antibody, Abcam, USA), and anti-Calnexin (1 : 2000, rabbit polyclonal antibody, Abcam, USA) antibodies. The characterization of the sEVs was performed following the Minimal Information for Studies of Extracellular Vesicles 2018 (MISEV2018) recommended by the International Society for Extracellular Vesicles (ISEV) [23].

### 2.4. Cell Proliferation Assay

The cells were seeded in 96-well plates at 2 × 10^3^ cells/well and treated with culture medium containing nBMMSCs-sEVs (50 *μ*g/mL), oBMMSCs-sEVs (50 *μ*g/mL), or PBS at the same volume. Two days later, proliferation was measured by the Cell Counting Kit-8 (CCK-8; Dojindo, Kumamoto, Japan). For quantitative analysis of cell proliferation, 10 *μ*L of the CCK-8 solution was added to each well. The absorbance was measured at 450 nm with a spectrophotometer.

### 2.5. Tube Formation Assay

Human umbilical vein endothelial cells (HUVECs) were purchased from the American Type Culture Collection (ATCC; Manassas, VA, USA). To evaluate the effect of nBMMSCs-sEVs and oBMMSCs-sEVs on HUVEC tube formation, HUVECs were seeded at 2 × 10^4^ cells/well in Matrigel-coated 96-well plates. The cells were cultured in culture medium supplemented with nBMMSCs-sEVs (50 *μ*g/mL), oBMMSCs-sEVs (50 *μ*g/mL), or PBS at the same volume. Three hours later, tube formation was observed under an optical microscope. Tube-forming structures were analyzed by counting the number of complete tubes using ImageJ software.

### 2.6. Enzyme-Linked Immunosorbent Assay (ELISA)

To determine the levels of vascular endothelial growth factor (VEGF) secreted by HUVECs, the cells were seeded in 6-well plates and cultured in culture medium containing nBMMSCs-sEVs (50 *μ*g/mL), oBMMSCs-sEVs (50 *μ*g/mL), or PBS at the same volume. Two days later, the cell supernatant was collected and centrifuged to remove the cellular debris. The concentrations of VEGF were tested by using a Human VEGF Quantikine ELISA Kit (R&D Systems, Inc., MN, USA) according to the manufacturer's instructions.

### 2.7. Alkaline Phosphatase (ALP) Activity Assay and Alizarin Red Staining

To assess osteogenic differentiation, BMMSCs were cultured with osteogenic medium (*α*-MEM containing 10% exosome-free FBS, 10 nM dexamethasone, 50 *μ*M L-ascorbic acid-2-phosphate, and 10 mM *β*-glycerophosphate; all from Sigma) supplemented with nBMMSCs-sEVs (50 *μ*g/mL), oBMMSCs-sEVs (50 *μ*g/mL), or PBS at the same volume. The medium was changed every three days. To evaluate osteogenic differentiation, the ALP activity assay and alizarin red staining were performed according to a previously reported protocol [27]. After 7 days of osteogenic induction, the ALP activity was determined using p-nitrophenyl phosphate (p-NPP, Sigma) as substrate and colorimetrically quantified at 405 nm. After 14 days of osteogenic induction, alizarin red staining (ARS) was performed. The cells were fixed with 4% paraformaldehyde for 15 min, washed twice with distilled water, and stained with alizarin red for 30 min. For the quantitative assessment, alizarin red staining was desorbed with 10% cetylpyridinium chloride (Sigma), and the optical density was measured at 590 nm using a spectrophotometer (Thermo). The values of all the samples were normalized to the total concentration of the cellular proteins, which was measured using the BCA protein assay kit (Pierce, USA).

### 2.8. Immunofluorescence (IF)

After seeding into 24-well plates, the BMMSCs were treated with culture medium containing nBMMSCs-sEVs (50 *μ*g/mL), oBMMSCs-sEVs (50 *μ*g/mL), or PBS at the same volume. Two days later, the expression level and location of *β*-catenin were measured by immunofluorescence. Briefly, the cells were fixed for 10 min in 4% paraformaldehyde, permeabilized for 10 min with 0.25% Triton X-100, and blocked for 30 min with 1% bovine serum albumin. Then, the cells were incubated with an anti-*β*-catenin antibody (1 : 200, rabbit monoclonal antibody, Abcam, USA) overnight at 4°C and visualized with a Cy3-conjugated secondary antibody (1 : 1000, goat anti-rabbit (Cy3)-conjugated antibody, Abcam, USA). After washing the cells twice with PBS, the slides were counterstained with 4′,6-diamidino-2-phenylindole (DAPI) to stain the cell nuclei. The florescence was observed by a fluorescence microscope (Fluoview 500, Olympus, Japan).

### 2.9. Quantitative Real-Time Polymerase Chain Reaction (qRT-PCR) Analysis

After treatment with nBMMSCs-sEVs (50 *μ*g/mL), oBMMSCs-sEVs (50 *μ*g/mL), or PBS at the same volume for 2 days, the mRNA levels of VEGFA in the HUVECs and *β*-catenin in the BMMSCs were determined by qRT-PCR. After 14 days of osteogenic induction, the mRNA levels of Runt-related transcription factor 2 (RUNX2) and osteocalcin (OCN), which are important osteogenic markers, were detected by qRT-PCR. The total RNA was extracted with TRIzol reagent according to the instructions (Invitrogen, Carlsbad, CA, USA). RNA was reverse transcribed to cDNA using the PrimeScript RT Master Mix Kit (TaKaRa, Shiga, Japan). The levels of mRNA were measured by the SYBR Premix Ex Taq™ Kit (TaKaRa, Shiga, Japan). GAPDH served as an internal control. The following primers were used: VEGFA, forward: 5′-CGCTCGGTGCTGGAATTTGA-3′, reverse: 5′-AGTGGGGAATGGCAAGCAAA-3′; *β*-catenin, forward: 5′-CTTACGGCAATCAGGAAAGC-3′, reverse: 5′-TAGAGCAGACAGACAGCACCTT-3′; OCN, forward: 5′-ACCCTCTCTCTGCTCACTCTGCT-3′, reverse: 5′-GCTCCAACTCCATTGTTGAGGTAG-3′; RUNX2, forward: 5′-CTTCGTCAGCGTCCTATCAGTTC-3′, reverse: 5′-CAGCGTCAACACCATCATTCTG-3′; and GAPDH, forward: 5′-TATGACTCTACCCACGGCAAGT-3′, reverse: 5′-ATACTCAGCACCAGCATCACC-3′. Each sample was analyzed in triplicate.

### 2.10. Statistical Analysis

All the experiments were repeated in triplicate. All the data are displayed as the means ± SEMs. The unpaired two-tailed Student's *t*-test was applied to compare two groups with SPSS 18.0. All the data demonstrated a normal distribution and similar variation between groups checked by the Shapiro-Wilk test and Levene's test, respectively. A *p* value less than 0.05 was considered significant.

## 3. Results

### 3.1. Characterization of BMMSCs and sEVs

After the initial seeding, the BMMSCs rapidly expanded into colonies of confluent, spindle-shaped cells. The cell surface marker analysis (data not shown) by flow cytometry indicated that the cells were positive for CD29 (90.2%) and CD90 (95.4%) and negative for CD45 (0.73%). The cultured cells were thus considered to be BMMSCs.

**Figure 1:**
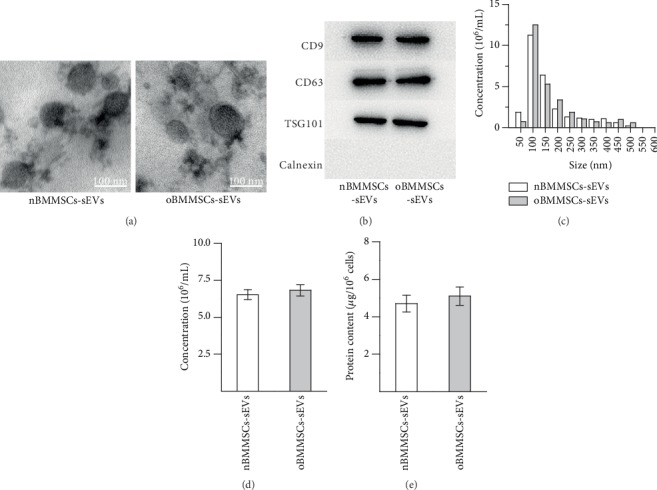
Characterization of sEVs derived from normal BMMSCs and ONFH BMMSCs. (a) Representative morphology of the sEVs as observed by transmission electron microscopy. (b) Detection of CD9, CD63, TSG101, and Calnexin expression by Western blotting. (c) Size distribution of the sEVs derived from normal BMMSCs and ONFH BMMSCs detected by NTA. (d) Concentrations of the sEVs derived from normal BMMSCs and ONFH BMMSCs detected by NTA. (e) Protein content in the sEVs derived from normal BMMSCs and ONFH BMMSCs. The results are from three independent experiments. The data are expressed as the means ± SEMs.

TEM, Western blotting, and nanoparticle tracking analysis were used to characterize the particles derived from normal BMMSCs and ONFH BMMSCs. As shown in Figure 1(a), the TEM images indicated that both nBMMSCs-sEVs and oBMMSCs-sEVs exhibited spheroidal morphology, and the size of these nanoparticles was 40–150 nm. Western blotting analysis indicated that nBMMSCs-sEVs and oBMMSCs-sEVs expressed exosomal markers, including CD9, CD63, and TSG101 (Figure 1(b)). In addition, neither nBMMSCs-sEVs nor oBMMSCs-sEVs expressed Calnexin, which is an endoplasmic reticulum membrane marker expressed in cells but less in sEVs. To analyze the counts and the size distribution of the particles derived from normal BMMSCs and ONFH BMMSCs, NTA was performed. The NTA results exhibited that nBMMSCs-sEVs and oBMMSCs-sEVs showed similar concentrations with similar size distributions (Figures 1(c) and 1(d)). The protein content in the sEVs was quantified by a BCA assay, and the results showed no marked difference between the two groups (Figure 1(e)). Taken together, these results indicated that the sEV preparations in the present study included exosomes.

### 3.2. Effects of sEVs on BMMSC Proliferation and Osteogenic Differentiation Were Attenuated in Steroid-Induced Osteonecrosis of the Femoral Head

**Figure 2:**
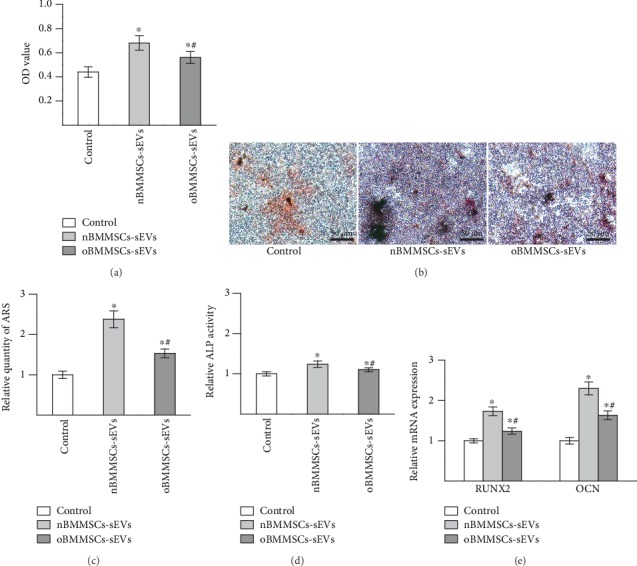
The effects of sEVs on BMMSC proliferation and osteogenic differentiation were partially attenuated in steroid-induced osteonecrosis of the femoral head. (a) Proliferation of BMMSCs cultured with nBMMSCs-sEVs and oBMMSCs-sEVs as detected by CCK-8 assay. (b) Assessment of mineralization by alizarin red staining. (c) Quantitative analysis of alizarin red staining. (d) Quantification of ALP activity. (e) Expression of RUNX2 and OCN mRNA was measured by qRT-PCR. The results are from three independent experiments. The data are expressed as the means ± SEMs. ^∗^*p* < 0.05 compared with the control group as determined by Student's *t*-test. ^#^*p* < 0.05 compared with the nBMMSCs-sEVs treatment group as determined by Student's *t*-test.

The proliferation of BMMSCs was detected by the CCK-8 assay (Figure 2(a)). The results showed that compared with the control group, both nBMMSCs-sEVs and oBMMSCs-sEVs promoted BMMSC proliferation (*p* < 0.05). Moreover, BMMSCs cultured with oBMMSCs-sEVs showed reduced proliferation compared with those cultured with nBMMSCs-sEVs (*p* < 0.05). Calcium deposition and ALP activity were investigated to estimate osteogenic differentiation. Calcium deposition was examined by alizarin red staining (Figures 2(b) and 2(c)). The results showed that both BMMSCs cultured with nBMMSCs-sEVs and oBMMSCs-sEVs showed enhanced mineralization (*p* < 0.05), and the oBMMSCs-sEVs group showed reduced mineralization compared with the nBMMSCs-sEVs group (*p* < 0.05). Similarly, the results of the ALP activity analysis (Figure 2(d)) demonstrated that BMMSCs cultured with either nBMMSCs-sEVs or oBMMSCs-sEVs showed increased ALP activity compared with BMMSCs cultured with the control treatment (*p* < 0.05). BMMSCs cultured with oBMMSCs-sEVs showed lower ALP activity than BMMSCs cultured with nBMMSCs-sEVs (*p* < 0.05). Moreover, we examined the effects of nBMMSCs-sEVs and oBMMSCs-sEVs on the mRNA expression of RUNX2 and OCN by qRT-PCR. The results (Figure 2(e)) indicated that both nBMMSCs-sEVs and oBMMSCs-sEVs increased the mRNA levels of RUNX2 and OCN (*p* < 0.05). BMMSCs cultured with oBMMSCs-sEVs showed lower mRNA levels of RUNX2 and OCN than BMMSCs cultured with nBMMSCs-sEVs (*p* < 0.05). Taken together, these results indicated that sEVs derived from both normal BMMSCs and ONFH BMMSCs can promote the osteogenesis of BMMSCs in vitro. However, the osteogenic potential of the sEVs obtained from ONFH BMMSCs was partially attenuated compared with that of the sEVs derived from normal BMMSCs.

### 3.3. Effects of sEVs on HUVEC Proliferation, VEGF Expression, and Tube Formation Were Attenuated in Steroid-Induced Osteonecrosis of the Femoral Head

**Figure 3:**
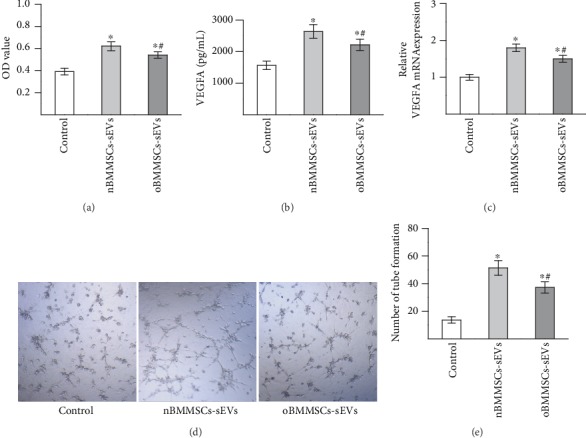
Effects of sEVs on HUVEC proliferation, VEGF expression, and tube formation were partially attenuated in steroid-induced osteonecrosis of the femoral head. (a) Proliferation of HUVECs cultured with nBMMSCs-sEVs and oBMMSCs-sEVs as detected by CCK-8 assay. (b) Secretion levels of VEGF as measured by ELISA. (c) Expression levels of VEGFA were measured by qRT-PCR. (d) Tube formation by HUVECs treated with nBMMSCs-sEVs and oBMMSCs-sEVs. (e) Quantitative analysis of the tube formation assay. The results are from three independent experiments. The data are expressed as the means ± SEMs. ^∗^*p* < 0.05 compared with the control group as determined by Student's *t*-test. ^#^*p* < 0.05 compared with the nBMMSCs-sEVs treatment group as determined by Student's *t*-test.

As endothelial cell proliferation, angiogenic factor expression, and tube formation are key processes in angiogenesis, we investigated the effects of nBMMSCs-sEVs and oBMMSCs-sEVs on HUVEC proliferation, VEGF expression, and tube formation. The proliferation of HUVECs was studied by the CCK-8 assay (Figure 3(a)). The CCK-8 assay demonstrated that HUVECs cultured with either nBMMSCs-sEVs or oBMMSCs-sEVs showed enhanced proliferation of HUVECs compared with HUVECs cultured with the control treatment (*p* < 0.05). HUVECs cultured with oBMMSCs-sEVs showed reduced proliferation than those cultured with nBMMSCs-sEVs (*p* < 0.05). The expression level of VEGF, which is critical for angiogenesis, was measured by ELISA and qRT-PCR after the HUVECs were cultured with nBMMSCs-sEVs or oBMMSCs-sEVs for 2 days. As shown in Figure 3(b), the ELISA results indicated that HUVECs cultured with either nBMMSCs-sEVs or oBMMSCs-sEVs induced higher VEGF secretion by HUVECs than HUVECs cultured with control treatment (*p* < 0.05). However, HUVECs cultured with oBMMSCs-sEVs showed lower VEGF secretion than HUVECs cultured with nBMMSCs-sEVs (*p* < 0.05). As shown in Figure 3(c), the qRT-PCR results indicated that both nBMMSCs-sEVs and oBMMSCs-sEVs increased the mRNA level of VEGFA in HUVECs (*p* < 0.05). HUVECs cultured with nBMMSCs-sEVs showed higher mRNA levels of VEGFA than HUVECs cultured with oBMMSCs-sEVs (*p* < 0.05).

The effects of nBMMSCs-sEVs and oBMMSCs-sEVs on the tube formation of HUVECs were investigated by a three-dimensional Matrigel assay (Figures 3(d) and 3(e)). Compared with the control treatment, both nBMMSCs-sEVs and oBMMSCs-sEVs markedly promoted the formation of capillary-like structures in HUVECs. Quantitative analysis showed that the number of tubes formed was lower in the oBMMSCs-sEVs group than in the nBMMSCs-sEVs group, indicating that the potential effects of oBMMSCs-sEVs on tube formation were partially attenuated. Taken together, these results demonstrated that both sEVs derived from normal BMMSCs and sEVs derived from ONFH BMMSCs can promote angiogenesis. However, the angiogenic potential of sEVs derived from ONFH BMMSCs was partially attenuated.

### 3.4. Effects of nBMMSCs-sEVs and oBMMSCs-sEVs on the Wnt/*β*-Catenin Pathway in BMMSCs

**Figure 4:**
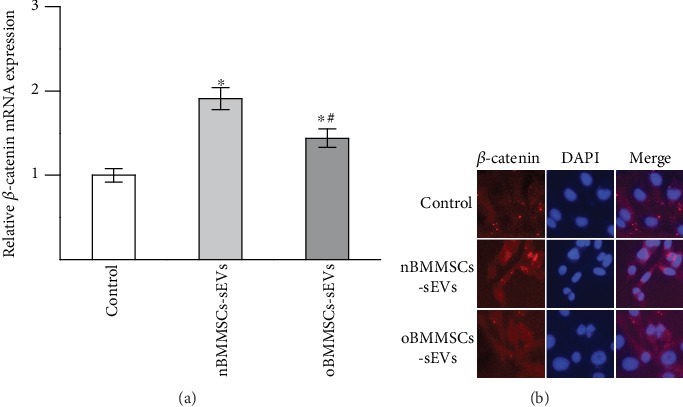
Effects of nBMMSCs-sEVs and oBMMSCs-sEVs on *β*-catenin expression in BMMSCs. (a) mRNA level of *β*-catenin was measured by qRT-PCR. (b) Protein level of *β*-catenin in BMMSCs as detected by immunofluorescence staining. The *β*-catenin protein was detected with a red color using a secondary antibody, and DAPI nuclear staining is shown in blue. The results are from three independent experiments. The data are expressed as the means ± SEMs. ^∗^*p* < 0.05 compared with the control group as determined by Student's *t*-test. ^#^*p* < 0.05 compared with the nBMMSCs-sEVs treatment group as determined by Student's *t*-test.

The Wnt/*β*-catenin pathway plays important roles in the regulation of bone metabolism and is regarded as the master moderator of BMMSC differentiation. If activated, this pathway contributes to osteogenic differentiation. We investigated the effects of nBMMSCs-sEVs and oBMMSCs-sEVs on *β*-catenin expression in BMMSCs by qRT-PCR and immunofluorescence analysis. The qRT-PCR results showed that the mRNA expression of *β*-catenin increased in the nBMMSCs-sEVs group and oBMMSCs-sEVs group compared with that in the control group, and the expression of *β*-catenin in the oBMMSCs-sEVs group was decreased compared with that in the nBMMSCs-sEVs group (Figure 4(a)). The immunofluorescence analysis showed similar results. The fluorescence intensity of *β*-catenin in the nBMMSCs-sEVs group and oBMMSCs-sEVs group increased compared with that in the control group, and the fluorescence intensity was inhibited in the oBMMSCs-sEVs group compared with that in the nBMMSCs-sEVs group (Figure 4(b)).

## 4. Discussion

In the present study, we found that the proosteogenic and proangiogenic ability of sEVs derived from BMMSCs from rats with steroid-induced ONFH was partially attenuated. Our results showed that both sEVs secreted by normal BMMSCs and sEVs secreted by ONFH BMMSCs promoted the proliferation and osteogenic differentiation of BMMSCs and enhanced the angiogenesis of HUVECs in vitro. However, sEVs derived from ONFH BMMSCs had an attenuated effect compared with sEVs derived from normal BMMSCs, indicating that the proosteogenic and proangiogenic effects of sEVs were partially attenuated in steroid-induced ONFH.

Defective characteristics of BMMSCs have been previously reported in patients and animals with steroid-induced ONFH [3–6, 28]. ONFH impairs the proliferation and osteogenic differentiation of BMMSCs. However, the main causes of these defects in BMMSCs are not completely understood. Various studies have shown that BMMSCs produce regeneration effects via the secretion of paracrine factors. Among these paracrine factors, sEVs possess therapeutic functions similar to those of BMMSCs. In addition, the biological activities of sEVs can reflect the biological characteristics of their source cells. To date, the biological activities of sEVs derived from BMMSCs from individuals with steroid-induced ONFH have not been investigated. Hence, we investigated the ability of sEVs derived from steroid-induced ONFH BMMSCs and found that there was a deficiency in the function of these sEVs. The defect in the function of the sEVs derived from steroid-induced ONFH may be related to the attenuated characteristics of the BMMSCs in steroid-induced ONFH.

Increasing studies have demonstrated that sEVs secreted by various stem cells can promote the osteogenesis of BMMSCs. Takeuchi et al. showed that exosomes secreted by BMMSCs enhance cellular migration, osteogenic differentiation, and angiogenic gene expression in mesenchymal stem cells [29]. Zhu et al. found that exosomes secreted by BMMSCs promote proliferation and migration, induce osteogenic differentiation, and enhance osteogenic gene expression in BMMSCs [30]. The results of our study similarly suggested that the sEVs secreted by BMMSCs promoted BMMSC proliferation, osteogenic differentiation, and osteogenic gene expression. Qi et al. investigated the effects of exosomes derived from human-induced pluripotent stem cell-derived mesenchymal stem cells (hiPSC-MSCs) on the osteogenic differentiation and proliferation of BMMSCs obtained from ovariectomized rats [16]. They found that exosomes derived from hiPSC-MSCs promote the osteogenic differentiation and proliferation of BMMSCs obtained from ovariectomized rats. Fang et al. examined the effect of exosomes derived from normal BMMSCs on the osteogenic differentiation of BMMSCs derived from rats with steroid-induced ONFH [31]. The results revealed that the exosomes derived from normal BMMSCs induce the osteogenic differentiation and inhibit the adipogenic differentiation of BMMSCs derived from rats with steroid-induced ONFH. In addition, it has been demonstrated that sEVs secreted by various stem cells can promote the angiogenesis of HUVECs. Liang et al. proved that exosomes derived from adipose-derived MSCs could significantly promote proangiogenic gene expression and tube formation in HUVECs [32]. Hu et al. showed that exosomes obtained from hiPSC-MSCs could promote the migration, proliferation, and tube formation of HUVECs and activate the expression of proangiogenic molecules [13]. In the present study, we found that sEVs obtained from BMMSCs accelerated the proliferation, VEGF expression, and tube formation of HUVECs. Similarly, Nakamura et al. suggested that exosomes derived from mesenchymal stem cells promote the tube formation and migration of HUVECs [33].

Importantly, the proosteogenic and proangiogenic effects of EVs in the present study were not exclusively exerted by exosomes, since the particles obtained were mixed EVs including exosomes and microvesicles and microvesicles also have been reported to promote angiogenesis and tissue repair [34–36]. It cannot be excluded that microvesicles are responsible for the proosteogenic and proangiogenic effects.

Studies have indicated that pathological conditions affect the functions of exosomes [20–22]. Zhu et al. demonstrated that the proosteogenic and proangiogenic effects of exosomes secreted by BMMSCs are impaired in rats with type 1 diabetes [30]. To date, the effects of steroid-induced ONFH on sEVs derived from BMMSCs have not been investigated. Our results indicated that sEVs secreted by ONFH BMMSCs could also promote the proliferation and osteogenic differentiation of BMMSCs and enhance the proliferation, VEGF expression, and tube formation of HUVECs. However, the proosteogenic and proangiogenic effects of sEVs derived from ONFH BMMSCs were partially attenuated compared with those of sEVs derived from normal BMMSCs.

Recent studies have shown that sEVs derived from BMMSCs play significant roles in the treatment of various diseases, including myocardial infarctions, kidney injury, stroke, bone defects, and osteonecrosis of the femoral head [12, 14–17]. Takeuchi et al. confirmed that exosomes secreted by BMMSCs promote bone regeneration in vivo in a rat model of calvarial bone defects [29]. Similar to the results of Takeuchi et al., Zhu et al. indicated that exosomes secreted by BMMSCs promote bone regeneration and neovascularization in a rat model of calvarial defects [30]. Hu et al. proved that exosomes secreted by hiPSC-MSCs attenuate limb ischaemia by promoting angiogenesis in a mouse model of hindlimb ischaemia [13]. Qi et al. found that exosomes secreted by hiPSC-MSCs promote the repair of osteoporotic bone defects by enhancing osteogenesis and angiogenesis in a rat model of critical-sized bone defects [16]. Liu et al. revealed that the administration of exosomes secreted by hiPSC-MSCs significantly prevents bone loss and enhances angiogenesis in the femoral head in a rat model of steroid-induced ONFH [17]. However, most of the sEVs used in the above studies were derived from normal BMMSCs. The use of sEVs derived from ONFH BMMSCs to induce osteogenesis and angiogenesis has not been reported. Based on our in vitro results, which indicated that the bone regenerative potential and proangiogenic effects of sEVs derived from ONFH BMMSCs were attenuated, the efficacy of the transplantation of sEVs derived from ONFH BMMSCs to promote bone regeneration and angiogenesis might be compromised.

It is generally accepted that sEVs accelerate tissue repair and regeneration by transferring proteins, RNAs, and other biological factors to recipient cells, thereby regulating related pathways in the recipient cells. Anderson et al. comprehensively characterized the proteinaceous contents of MSC-derived exosomes and revealed that exosomes derived from MSCs induce angiogenesis in endothelial cells via nuclear factor-kappa B signaling [37]. Kuang et al. found that exosomes secreted by Wharton's jelly of human umbilical cord mesenchymal stem cells inhibit the apoptosis of osteocytes and prevent ONFH through the miR-21-PTEN-AKT signaling pathway in a rat model of steroid-induced ONFH [38]. Liu et al. demonstrated that exosomes secreted by iPS-MSCs can induce activation of the PI3K/AKT signaling pathway in endothelial cells [17]. Ma et al. suggested that exosomes secreted by adipose-derived stem cells promote cutaneous wound healing by activating the Wnt/*β*-catenin signaling pathway [39]. Zuo et al. indicated that exosomes secreted by BMMSCs attenuate radiation-induced bone loss by activating the Wnt/*β*-catenin pathway [40]. The Wnt/*β*-catenin signaling pathway is regarded as the master moderator of BMMSCs and has crucial effects on osteogenic and adipogenic differentiation [41]. In our study, we found that the protein and mRNA expression of *β*-catenin in BMMSCs was increased after treatment with sEVs. Furthermore, we demonstrated that sEVs induced VEGF secretion and promoted VEGFA mRNA expression in HUVECs. These results might partially elucidate the mechanisms of the proangiogenic and proosteogenic effects of sEVs. However, the levels of *β*-catenin and VEGF expression were different between the normal BMMSC-derived sEV treatment group and the ONFH BMMSC-derived sEV treatment group. These findings suggest that differentially expressed signaling components might play critical roles in the attenuated proosteogenic and proangiogenic effects of sEVs derived from ONFH BMMSCs.

The present study has limitations. One limitation is that the number of replicates was small (*n* = 3) in our study. In addition, the exact mechanisms of the attenuated proosteogenic and proangiogenic effects of the sEVs derived from ONFH BMMSCs have not been investigated. Furthermore, the detailed molecular differences between the components in sEVs derived from ONFH BMMSCs and in sEVs derived from normal BMMSCs have not been elucidated. Thus, further studies are needed. In the near future, we will conduct additional studies to compare the contents of the sEVs derived from normal BMMSCs and ONFH BMMSCs and clarify the detailed mechanisms.

In summary, the present study suggests that the proosteogenic and proangiogenic effects of sEVs derived from ONFH BMMSCs were partially attenuated compared with those of sEVs derived from normal BMMSCs. This study may provide a better understanding of the selection of source cells for sEV therapy in the future.

## Data Availability

The datasets supporting the conclusions of this article are included within the article and available from the corresponding author on reasonable request.

## Abbreviations

ALP: Alkaline phosphatase activity.
ARS: Alizarin red staining.
ATCC: American Type Culture Collection.
BMMSCs: Bone marrow mesenchymal stem cells.
CCK-8: Cell Counting Kit-8.
EPCs: Endothelial progenitor cells.
EVs: Extracellular vesicles.
FBS: Fetal bovine serum.
HUVECs: Human umbilical vein endothelial cells.
ISEV: International Society for Extracellular Vesicles.
LPS: Lipopolysaccharide.
MVBs: Multivesicular bodies.
nBMMSCs-sEVs: sEVs secreted by BMMSCs derived from normal rats.
NTA: Nanoparticle tracking analysis.
oBMMSCs-sEVs: sEVs secreted by BMMSCs derived from steroid-induced ONFH rats
OCN: Osteocalcin.
OD: Optical density.
ONFH: Osteonecrosis of the femoral head.
p-NPP: p-Nitrophenyl phosphate.
RUNX2: Runt-related transcription factor 2.
sEVs: Small extracellular vesicles.
TEM: Transmission electron microscopy.
*α*-MEM: *α*-Minimal essential medium.
